# Examination of a partial dietary self‐monitoring approach for behavioral weight management

**DOI:** 10.1002/osp4.416

**Published:** 2020-04-26

**Authors:** Deborah F. Tate, Danika A. Quesnel, Lesley Lutes, Karen E. Hatley, Brooke T. Nezami, Alexis C. Wojtanowski, Angela M. Pinto, Julianne Power, Molly Diamond, Kristen Polzien, Gary Foster

**Affiliations:** ^1^ Department of Nutrition University of North Carolina at Chapel Hill Chapel Hill North Carolina USA; ^2^ Department of Health Behavior University of North Carolina at Chapel Hill Chapel Hill North Carolina USA; ^3^ Lineberger Comprehensive Cancer Center University of North Carolina at Chapel Hill Chapel Hill North Carolina USA; ^4^ WW New York NY USA; ^5^ Department of Psychology University of British Columbia, Okanagan Campus Kelowna British Columbia Canada; ^6^ Center for Weight and Eating Disorders, Perelman School of Medicine University of Pennsylvania Philadelphia Pennsylvania USA; ^7^ Psychology Baruch College New York City New York USA

**Keywords:** cravings, diet, self‐monitoring, weight

## Abstract

**Introduction:**

Dietary self‐monitoring in behavioral weight loss programmes traditionally involves keeping track of all foods and beverages to achieve a calorie deficit. While effective, adherence declines over time. WW™ (formerly Weight Watchers), a widely available commercial weight management programme, sought to pilot an approach that permitted participants to consume over 200 foods without monitoring them.

**Methods:**

The current study used a pre‐post evaluation design with anthropometric, psychosocial and physical health assessments at baseline, 3 and 6 months.

**Results:**

Participants (*N* = 152) were, on average, 48.4 (±12.3) years old, with body mass index (BMI) of 32.8 (±4.8) m/kg^2^ and 94% female. Mean weight loss was 6.97 + 5.55 kg or 7.9 ± 6.1% of initial body weight (*p*s < .0001) at 6 months. One third (32.6%) of the sample lost 10% or more of initial body weight. Significant improvements in hunger, cravings, happiness, sleep, quality of life, aerobic stamina, flexibility and blood pressure were observed. Attendance at group meetings, as well as decreases in hunger, and fast food cravings from baseline to 3 months were associated with achieving 10% weight loss at 6 months (*p* < .01).

**Conclusions:**

Using an approach that does not require self‐monitoring of all foods and beverages produced significant weight losses and other physical and psychosocial improvements.

## 1 INTRODUCTION

Structured behavioral weight loss programmes are effective at producing clinically significant weight loss (5%–10%) over time^1^ and result in reductions in co‐morbid illnesses.^1^, ^2^ These interventions commonly prescribe an energy deficit in the form of a total daily calorie intake goal, combined with dietary self‐monitoring, to ensure adherence to the prescription.^2^, ^3^ Self‐monitoring, a key concept in self‐regulation, is a series of measurements, observations and recordings that enhance awareness^4^ and, when applied to diet, involves measuring and recording all foods and beverages consumed along with other metrics such as the time eaten, their calorie content, and at times, hunger or mood before eating. While self‐monitoring is effective and has long been identified as a key predictor of weight loss success, studies demonstrate that dietary self‐monitoring decreases over time, subsequently leading to suboptimal outcomes.^2^, ^3^, ^5^

The effort involved in monitoring all foods, portions and calories or other metrics is substantial and relates directly to a decline in dietary programme adherence.^5^ Additionally, reducing calories without attention to nutrient composition may lead to hunger and dissatisfaction and may also result in suboptimal weight change.^1^ Certain protein‐rich, low‐fat foods can help improve satiety and combat food cravings.^6^ Additionally, dietary approaches with a greater allotment of low‐energy‐dense foods such as fruits and vegetables, which allow individuals to consume satisfying portions of food, have shown a positive effect on weight loss.^7^ Therefore, an approach that promotes the adoption of an energy‐reduced diet while also reducing the burden of self‐monitoring is a well‐reasoned and potentially sustainable approach for weight management but has not been well studied to date.

WW^TM^ (formerly Weight Watchers) is an empirically validated, globally available weight management programme.^8^, ^9^, ^10^ In the current pilot study, the efficacy and acceptability of a modified food plan for weight loss that allowed reduced self‐monitoring, delivered within the context of the WW workshop + digital programme (includes in‐person workshops and access to digital tools), were examined in a 6‐month pre‐post design. The objective of the current study was to test the efficacy of this WW food plan for producing 6‐month weight loss and to determine predictors of achieving a 10% weight loss. The primary outcome was weight loss at 6 months in kilograms (and expressed as percent of initial body weight lost at 6 months). Secondary outcomes included percent of individuals reaching 3%, 5% and 10% weight loss at 6 months, as well as other physical outcomes of aerobic stamina, flexibility and blood pressure. We included exploratory measures that might be affected by the 200 zero‐point food plan such as feelings of hunger, fullness and food cravings. Finally, we included psychosocial correlates of weight loss that are not well studied in commercial programs to date, including sleep quality, quality of life, and happiness to examine changes associated with this magnitude of weight loss.

## 2 MATERIALS AND METHODS

### 2.1 Study design and recruitment

This was a single‐arm, prospective, pre‐post evaluation of a 6‐month weight loss intervention. Recruitment took place over the course of 4 weeks in January and February 2017 via social media, emails to university listservs and emails to former WW members. Interested individuals were required to meet the following inclusion criteria: male or female age 18–75 years, body mass index (BMI) between 25 and 43 m/kg^2^, report that they wanted to lose weight, willing to discontinue over‐the‐counter dietary supplements other than a multivitamin, willing to follow recommendations of the protocol, state their demographic information, able to use a smartphone with adequate programming (iOS 8.0 or later and 600MB of available storage) to use the WW app and commit to attending 24 weekly group sessions for the WW programme. Individuals were excluded from the study if they had been a member of WW in the past 12 months. Eligible individuals were invited to attend an in‐person orientation session at the University of North Carolina (UNC) Weight Research Program clinic, where study staff explained the study procedures and intervention. Baseline physical measures were collected at a subsequent visit, and questionnaires were completed online using REDCap, a secure online system for administering surveys. The study was approved by the Non‐Biomedical Institutional Review Board at the UNC at Chapel Hill. Enrolled participants provided written informed consent.

### 2.2 Intervention

Study participants received the standard WW programme available to other WW community participants with the exception of the modified food plan. The programme consists of three pillars—food, activity and mindset—and emphasizes behavioral skills and techniques. Participants chose their own weight goal.

The intervention was delivered in 30‐ to 60‐min weekly workshops led by WW coaches at the UNC research centre location. WW coaches were existing employees of WW living and working in the community local to the research centre site. They were identified by the local territory manager to participate in the study. Coaches in WW are members who have lost weight themselves on the programme and regularly receive webinars and instructions on new programme offerings by the company. For this study, coaches participated in a half‐day in‐person training that included an introduction to the plan being used in this research, guidance on running the meetings and considerations for implementing the programme within a clinical trial. Calls with WW team, coaches and research staff were held after Weeks 2, 4, 8, 12, 26, 20 and 24 to collect feedback. Fidelity was not assessed in a formal manner.

A member of the research team was on hand to answer study‐related questions and to facilitate access to the building; however, they did not deliver the intervention. In the workshops, WW coaches reviewed weekly progress, successes and barriers with participants, offered new behavioral skills through a semistructured interactive session, facilitated group discussions on the week's topic and provided guidance on the application of the new skills into real‐life settings. There was a scheduled topic each week of the programme, for example, thinking styles, responding to setbacks (behavioral and weight gains), planning ahead, overcoming barriers, social support mindful eating and distinguishing hunger from other reasons for eating. All participants downloaded a study‐specific WW app onto their smartphone that included diet and activity self‐monitoring and other resources such as recipes, meal ideas and topics related to weight management. Additionally, they received printed weekly take‐home skill builder worksheets and WW emails.

#### 2.2.1 Dietary goals

The core of the WW Food Programme is the SmartPoints® system, which is a method of self‐monitoring dietary intake. In the experimental Food Plan tested herein, over 200 foods including (but not limited to) skinless chicken and turkey breast, nonfat plain yogurt, eggs, fish, seafood, legumes and most fruits and vegetables were assigned a SmartPoints® value of zero (ZeroPoint foods) and did not require weighing, measuring or self‐monitoring. These foods were selected because they formed the foundation of a healthy eating pattern based on World Health Organization and USDA 2015–2020 Dietary Guidelines for Americans^11^, ^12^ and were considered low risk for overconsumption. A full listing of all 200 foods can be found in Table 1. Beyond these, the SmartPoints® system assigned each food and beverage a SmartPoints® value per volume based on four components: calories, sugar, saturated fat and protein. Foods higher in lean protein have lower SmartPoints values, while foods higher in calories, saturated fat and sugar have higher SmartPoints values. Participants self‐monitored their consumption in SmartPoints® with the study‐specific digital monitoring app.

**TABLE 1:** Zero‐point foods

Beans and legumes
Adzuki beans
Alfalfa sprouts
Bean sprouts
Black beans
Black‐eyed peas
Cannellini beans
Chickpeas
Edamame
Fava beans
Great Northern beans
Kidney beans
Lentils
Lima beans
Lupini beans
Navy beans
Pinto beans
Refried beans, canned, fat‐free
Soy beans
Chicken and turkey breast
Ground chicken breast
Ground turkey, 98% fat‐free
Ground turkey breast
Skinless chicken breast
Skinless turkey breast
Eggs
Egg substitute
Egg whites
Egg yolks eggs
Fish/shellfish
Abalone
Alaskan king crab
Anchovies, in water
Arctic char
Bluefish
Branzino
Butterfish
Canned tuna, in water
Carp
Catfish
Caviar
Clams
Cod
Crabmeat, lump
Crayfish
Cuttlefish
Dungeness crab
Eel
Fish roe
Flounder
Grouper
Haddock
Halibut
Herring
Lobster
Mahi
Monkfish
Mussels
Octopus
Orange roughy
Oysters
Perch
Pike
Pollock
Pompano
Salmon
Sardines, canned in water or sauce
Sashimi
Scallops
Sea bass
Sea cucumber
Sea urchin
Shrimp
Smelt
Smoked haddock
Smoked salmon
Smoked sturgeon
Smoked trout
Smoked whitefish
Snails
Snapper
Sole
Squid
Steelhead trout
Striped bass
Sturgeon
Swordfish
Tilapia
Trout
Tuna
Turbot
Wahoo
Whitefish
Fruits
Apples
Applesauce, unsweetened
Apricots, fresh
Bananas
Blackberries
Blueberries
Cantaloupe
Cherries
Clementines
Cranberries, fresh
Dragon fruit
Figs, fresh
Frozen mixed berries, unsweetened
Fruit cocktail, unsweetened
Fruit salad, unsweetened
Grapefruit
Grapes
Guava
Honeydew
Kiwi
Kumquats
Lemons
Limes
Mangoes
Meyer lemons
Nectarines
Oranges
Papayas
Peaches
Pears
Persimmons
Pineapples
Plums
Pomegranates
Pomelo
Raspberries
Star fruit
Strawberries
Tangerines
Watermelon
Nonfat yogurt and soy yogurt
Greek yogurt, plain, nonfat
Plain yogurt, nonfat
Quark, plain, up to 1% fat
Soy yogurt, plain
Tofu and tempeh
Firm tofu
Silken tofu
Smoked tofu
Soft tofu
Tempeh
Vegetables (starchy)
Canned corn
Corn
Green peas
Parsnips
Peas
Split peas
Succotash
Vegetables (nonstarchy)
Acorn squash
Artichoke hearts, no oil
Artichokes
Arugula
Asparagus
Baby corn
Bamboo shoots
Basil
Beet greens
Beets
Bok choy
Broccoli
Broccoli rabe
Broccoli slaw
Brussels sprouts
Butter/Bibb lettuce
Butternut squash
Cabbage
Canned pimientos
Carrots
Cauliflower
Cauliflower rice
Celery
Chives
Cilantro
Coleslaw mix
Collard greens
Cucumber
Eggplant
Endive
Escarole
Fennel
Frozen stir‐fry vegetables, no sauce
Garlic
Ginger
Green leaf lettuce
Hearts of palm
Iceberg lettuce
Jicama
Kale
Kohlrabi
Leeks
Mint
Mixed greens
Mushrooms
Mustard greens
Napa cabbage
Nori (seaweed)
Oak leaf lettuce
Okra
Onions
Oregano
Parsley
Pea shoots
Peppers
Pickles, unsweetened
Pico de gallo
Pumpkin
Pumpkin puree
Radishes
Red leaf lettuce
Romaine lettuce
Rosemary
Rutabaga
Salsa, fat‐free
Sauerkraut
Scallions
Shallots
Spaghetti squash
Spinach
String beans
Summer squash
Swiss chard
Tarragon
Thyme
Tomatillos
Tomato puree, canned
Tomato sauce, canned
Tomatoes
Turnips
Water chestnuts
Wax beans
Zucchini

#### 2.2.2 SmartPoints® budget

Based on the Mifflin St‐Jeor formula,^13^ which factors in age, sex, height and weight, a personalized SmartPoints® budget was calculated. The SmartPoints® budget consists of a daily target plus an extra allotment of weekly points for flexibility. Participants were free to allocate their SmartPoints® as they wished and were encouraged to self‐monitor their SmartPoints® in the WW app.

#### 2.2.3 FitPoints® goal

In addition to dietary goals and self‐monitoring, the programme included self‐monitoring of physical activity based on FitPoints®. Each activity is assigned a FitPoints value based on its duration, intensity and type. Participants received a personalized daily goal based on their baseline activity level and were encouraged to monitor their FitPoints® in the WW app.

### 2.3 Measures

Study measures were collected at baseline, 3 and 6 months. Participants received $25 at baseline, $50 at 3 months and $100 at 6 months for completing assessment procedures.

#### 2.3.1 Demographics and health history

At baseline only, participants provided demographic and health information, including age, gender, education, income, race/ethnicity, weight and smoking history.

#### 2.3.2 Anthropometric measurements

Weight was taken to the nearest 0.1 kg on a digital scale (Tanita BWB 800) while the participant was in light clothing without shoes. Height was measured on a wall‐mounted stadiometer to the nearest 0.1 cm. Waist circumference was measured at the height of the iliac crest. Two measurements were taken, with a third measurement taken if the initial two were not within a certain range of one another (within 0.2 kg for weight, 0.5 cm for height and 1.0 cm for waist circumference). Each of these measurements was recorded in accordance with the National Health and Nutrition Examination survey anthropometry procedures manual.^14^ Weight measurements at baseline and 6 months were used to calculate percent weight loss. BMI was calculated accordingly and reported in kg/m^2^.

#### 2.3.3 Other physical measures

Resting blood pressure was measured in seated position using a GE Dinamap ProCare 100 after a 5‐min rest; the average of two measures was used. The 6‐min walk test was used to measure aerobic stamina and was administered using a standardized protocol.^15^ This submaximal test has been used as a measure of aerobic endurance and functional mobility in adults with and without disease and has shown to be a reliable measure with an intraclass correlation coefficient of >.90.^16^ Subjects walked as far as possible in 6 min around a series of traffic cones placed on a level corridor with a course measuring 30.0 m in length, taking rest periods as needed. The total distance walked was recorded. Pulse was measured immediately before and after walking. Flexibility was measured using the classic sit and reach test.^17^ Three measurements were taken at each assessment, and the best score was used for analysis.

#### 2.3.4 Self‐reported variables

Feelings of hunger and fullness in the past week were assessed using the hunger visual analogue scale (HVAS),^18^ which uses three items to assess hunger, fullness after meals and general fullness and rated on a 100‐point scale (0 *= not at all* to 100 *= extremely*). Retrospective recall of hunger over the past week has been shown to correlate with average prospective daily ratings during the same time frame and to have adequate test–retest reliability.^19^ HVAS has been used in a similar manner in recent weight loss intervention trials.^20^ Food cravings were assessed using the 33‐item Food Cravings Inventory II^21^ in which cravings for high‐fat foods, sweets, carbohydrates, fast food and fruits and vegetables at the current moment are rated on a 5‐point Likert scale (1 *= never* to 5 *= always*/*almost every day*). Sleep quality and duration were measured with the Pittsburgh Sleep Quality Index (PSQI),^22^, ^23^ which is a 19‐item scale with a total summed score ranging from 0 to 21, with a score above 5 suggesting poor sleep quality. The Oxford Happiness Questionnaire^24^ is a 29‐item scale that measures broad personal happiness on a 6‐point Likert scale (1 *= strongly disagree* to 6 *= strongly agree*). Example items include ‘I feel that life is very rewarding’ and ‘I am always committed and involved’. Weight‐related quality of life was measured with the Impact of Weight on Quality of Life‐Lite^25^ questionnaire, which includes 31 items assessing one's perception of how weight affects day‐to‐day life, rated on a 5‐point Likert scale (1 *= never true* to 5 *= always true*). The measure includes five subscales: physical function, self‐esteem, sexual life, public distress and work.

Attendance was measured by recording the participant's attendance at the weekly WW meetings. A WW team member recorded the participant's WW meeting attendance.

### 2.4 Sample size

With 150 participants, this study was powered to detect a minimum effect size for weight loss of 0.33 at 24 weeks for using a two‐sided test with 95% power and a significance level of .05. This was based on a prior study of the WW online programme that detected a difference of 1.4 kg (SD = 3.6; *d* = 0.39) compared with a control group.^26^

### 2.5 Data analysis

Analyses were conducted using SAS 9.4 (SAS, Cary, NC). Descriptive characteristics were calculated for demographic characteristics and levels of primary and secondary outcomes at all assessment points. PROC MI (multiple imputation) was used to develop five data sets with data imputed for missing values using the Markov chain Monte Carlo procedure. Paired *t* tests evaluated weight change, by imputation, from baseline to 6 months and changes in secondary outcomes from baseline to 6 months. Logistic regression models were used to determine which demographic characteristics and 3‐month values of self‐reported variables, controlling for baseline levels, were predictors of 10% weight loss at 6 months. Each paired *t* test and logistic regression model was run by imputation, and then results were pooled across imputation sets using PROC MIANALYZE, which combines parameter estimates into a single set of statistics.

## 3 RESULTS

### 3.1 Participants

Characteristics of the study sample are presented in Table 2. Participants (*N* = 152) were, on average, 48.36 (SD = 12.27) years old with a mean BMI of 32.79 (SD = 4.84) kg/m^2^. The majority of the sample was female (94%), Caucasian (79.6%), and 75% had a college or advanced degree. A little over half (57%) were former (>1 year ago) WW members. The majority (64%) lived with a spouse or romantic partner, and nearly half (44%) had a child in the home. Participants reported a diverse number of weight loss strategies used in the past, with 35% reporting experience with a weight loss app. Retention at follow‐up assessments was 97.4% and 91.4% at 3 and 6 months, respectively. Participants attended an average of 16.91 (SD = 5.81) of the 24 WW weekly meetings (70.5%, SD = 24.2%).

**TABLE 2:** Baseline demographic characteristics (*N* = 152)

Variable	% (*n*)
Gender	
Female	94.1 (143)
Male	5.9 (9)
Age	
18–29	8.6 (13)
30–39	13.2 (20)
40–49	29.6 (45)
50–59	31.6 (48)
60–69	15.8 (24)
70+	1.3 (2)
Former WW participants	57 (87)
Race^a^	
White	79.6 (121)
Black	13.2 (20)
Asian	5.3 (8)
Other	2.6 (4)
Ethnicity	
Hispanic	8.6 (13)
Non‐Hispanic	91.4 (139)
Education	
<High school grad	1.3 (2)
High school grad or GED	3.9 (6)
Vocational or training school	3.9 (6)
Some college or associate	15.8 (24)
College graduate	44.7 (68)
Masters or doctoral degree	30.3 (46)
Annual income	
<$5000	0.7 (1)
$16–24 999	1.3 (2)
$25–34 999	2.6 (4)
$35–49 999	13.8 (21)
$50–74 999	24.3 (37)
$75–99 999	13.2 (20)
$100 000+	40.1 (61)
Don't know	3.9 (6)
Occupation^a^	
Full‐time job	71.7 (109)
Part‐time job	11.2 (17)
Full‐time student	6.6 (10)
Part‐time student	2.0 (3)
Other	12.5 (19)

a Categories are not mutually exclusive.

### 3.2 Weight change

**FIGURE 1:**
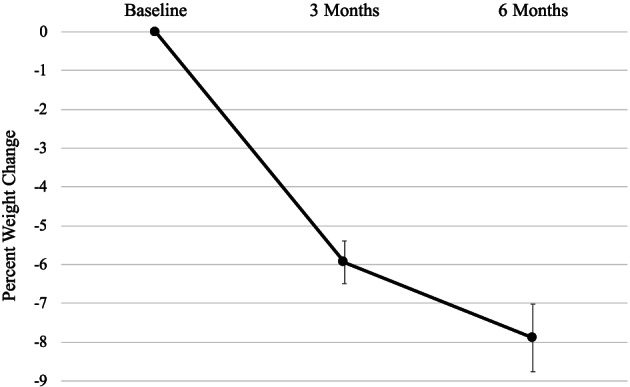
Mean percent weight change over time and 95% confidence intervals

**FIGURE 2:**
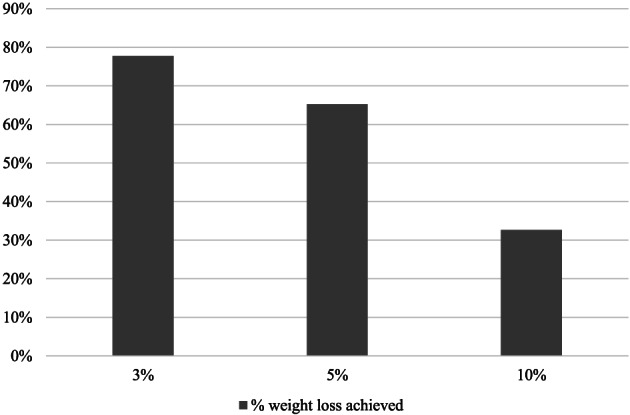
Percent of participants reaching 3%, 5% and 10% weight loss at 6 months

Participants lost 5.19 (SD = 3.44) kg at 3 months and 6.97 (SD = 5.55) kg at 6 months (*p*s < .0001), equivalent to 5.94% (SD = 3.93) and 7.89% (SD = 6.25) weight losses, respectively. For interpretation and comparability to other studies, Figure 1 shows percent weight change over time. Weight loss, while greater in the first 3 months of the programme, continued across the 6‐month study. At 6 months, 77.76% of participants had reached 3% weight loss, 65.26% reached 5% weight loss and 32.63% reached 10% weight loss (Figure 2).

### 3.3 Change in secondary outcomes

#### 3.3.1 Other physical measures

Table 3 presents means and standard deviations across time for physical measures. There were significant reductions in systolic and diastolic blood pressure and waist circumference and significant increases in flexibility and aerobic stamina (*p*s < .0001).

**TABLE 3:** Changes in physical measures using paired sample *t* test (MI)

	Mean (SD)			Mean (SD)	
	Baseline	3 months	Change	6 months	Change
Weight (kg)	88.36 (15.31)	83.15 (15.16)	**−5.21 (3.51)** ^***^	81.39 (15.40)	**−6.97 (5.68)** ^***^
BMI (kg/m^2^)	32.79 (4.84)	30.84 (4.73)	**−1.95 (1.32)** ^***^	30.19 (4.89)	**−2.60 (2.13)** ^***^
Blood pressure (mm/hg)					
Systolic	124.21 (12.20)	117.93 (13.55)	**−6.28 (11.61)** ^***^	116.32 (12.19)	**−7.89 (10.85)** ^***^
Diastolic	73.25 (8.87)	69.91 (8.89)	**−3.34 (7.54)** ^***^	69.02 (8.30)	**−4.23 (7.25)** ^***^
Waist circumference (cm)	107.44 (11.29)	103.36 (11.78)	**−4.08 (6.25)** ^***^	101.55 (12.24)	**−5.89 (7.11)** ^***^
Flexibility (sit and reach, in)	18.79 (4.02)	19.89 (3.79)	**1.10 (2.21)** ^***^	20.53 (3.92)	**1.74 (2.61)** ^***^
Aerobic stamina, distance (m)	517.44 (61.68)	535.86 (63.23)	**18.42 (35.65)** ^***^	549.09 (69.82)	**31.65 (47.31)** ^***^
Aerobic stamina, pulse (bpm)	114.12 (18.55)	102.07 (20.91)	**−12.05 (18.41)** ^***^	103.38 (21.35)	**−10.74 (20.21)** ^***^

Bolded values represent statistically significant changes.

*** *p* < .0001.

#### 3.3.2 Self‐reported variables

Table 4 presents means and standard deviations across time for self‐reported variables. Hunger was significantly lower at both 3 and 6 months compared with baseline (*p* < .05 and *p* < .001, respectively). Feelings of fullness after meals or in general were not significantly different from baseline at either follow‐up. Overall food cravings changed significantly between baseline, 3, and 6 months (*p*s < .0001). Cravings for high fats, sweets, starches and fast food were significantly lower at 6 months (*p* < .001), and fruit and vegetable cravings were significantly higher at 6 months compared with baseline (*p* < .05). Quality of life was improved at both 3 and 6 months (*p* < .001) as well as the following subscales: physical function, self‐esteem, sexual life (*p* < .0001) and work (*p* < .001). There were significant improvements in happiness from baseline to 3 and 6 months (*p* < .0001), and participants also reported improvements in sleep from baseline to 3 and 6 months (*p* < .05 and *p* < .001, respectively).

**TABLE 4:** Means (standard deviations) across time on self‐reported variables

	Baseline	3 months	Change Baseline—3	6 months	Change Baseline—6
Hunger and fullness					
Hunger	53.60 (19.40)	46.13 (21.10)	**−7.46 (25.71)** ^*^	43.66 (22.69)	**−9.94 (23.77)** ^**^
Fullness after meals	67.76 (15.97)	64.73 (15.90)	3.03 (20.31)	68.39 (16.94)	0.63 (21.85)
Fullness—general	61.86 (15.67)	59.54 (17.54)	2.32 (20.95)	64.56 (20.95)	2.70 (22.36)
Food cravings					
Food craving total	2.36 (0.49)	2.20 (0.47)	**−0.16 (0.47)** ^***^	2.15 (0.47)	**−0.20 (0.46)** ^***^
High fats	1.92 (0.55)	1.79 (0.55)	**−0.13 (0.45)** ^**^	1.75 (0.53)	**−0.17 (0.45)** ^***^
Sweets	2.64 (0.75)	2.36 (0.74)	**−0.27 (0.67)** ^***^	2.29 (0.74)	**0.35 (0.67)** ^***^
Carbohydrates	2.32 (0.64)	2.13 (0.61)	**0.19 (0.59)** ^**^	2.06 (0.61)	**−0.26 (0.57)** ^***^
Fast food	2.75 (0.75	2.53 (0.70)	**−0.22 (0.68)** ^***^	2.47 (0.77)	**−0.28 (0.69)** ^***^
Fruits and vegetables	2.35 (0.74)	2.44 (0.80)	0.09 (0.68)	2.47 (0.77)	**0.12 (0.68)** ^*^
PSQI total (sleep)^a^	5.90 (3.51)	5.27 (3.09)	**−0.62 (2.02)** ^*^	5.08 (3.32)	**−0.81 (2.41)** ^**^
Happiness	3.78 (0.40)	4.52 (0.65)	**0.74 (0.51)** ^***^	4.61 (0.70)	**0.83 (0.57)** ^***^
Impact of weight on quality of life					
Physical function	79.44 (17.11)	84.81 (15.26)	**5.37 (9.92)** ^***^	87.43 (16.40)	**7.99 (11.88)** ^***^
Self‐esteem	53.83 (26.71)	64.67 (25.28)	**10.83 (16.95)** ^***^	71.10 (25.54)	**17.27 (19.99)** ^***^
Sexual life	73.01 (28.99)	80.07 (25.49)	**7.06 (18.55)** ^***^	85.10 (22.20)	**12.09 (21.22)** ^***^
Public distress	91.55 (14.63)	92.93 (13.56)	1.39 (8.51)	93.12 (13.98)	1.58 (11.52)
Work	87.95 (16.87)	91.23 (14.84)	**3.27 (12.37)** ^**^	93.27 (15.13)	**5.32 (12.83)** ^**^
Total score	75.88 (16.30)	81.69 (15.61)	**5.78 (8.49)** ^***^	85.11 (15.87)	**9.23 (10.23)** ^**^

a Score of <5 considered ‘good’ sleep quality.

* *p* < .05.

** *p* < .001.

*** *p* < .0001.

### 3.4 Predictors of 10% weight loss

Table 5 presents the results evaluating factors associated with a 10% weight loss. Greater decreases in hunger from baseline to 3 months, greater attendance at workshops and greater decreases in fast food cravings from baseline to 3 months were associated with a higher likelihood of reaching 10% weight loss at 6 months. Demographics (age, gender, race and education), former participation in WW, changes in ratings of fullness, cravings of sweets, fats, carbohydrates, fruits and vegetables, sleep quality and happiness from baseline to 3 months were not associated with a 10% weight loss at 6 months.

**TABLE 5:** Predictors of 10% weight loss

	Parameter estimate	95% Cl	Sig.
Demographics			
Age	0.02	−0.01, 0.05	.19
Education (college degree vs. none)	−0.27	−1.08, 0.54	.51
Income ($50 000 or more vs. <$50 000)	−0.36	−1.35, 0.63	.48
Race			
White vs. Black	−0.91	−2.21, 0.38	.17
White vs. Other	−0.78	−2.41, 0.84	.35
Gender (male vs. female)	−0.54	−2.20, 1.12	.52
Self‐reported variables			
Hunger	−0.025	−0.029, −0.023	<.01
Full after meals	0.023	0.018, 0.030	.08
Full in general	0.020	0.018, 0.026	.09
Total food cravings	0.864	−0.964, −0.782	.07
Food cravings—fruits/vegetables	−0.266	−0.338, −0.211	.35
Food cravings—sweets	−0.319	−0.436, −0.264	.31
Food cravings—fast food fats	−0.903	−0.978, 0.805	<.01
Food cravings—fat	−0.609	−0.706, −0.534	.18
Food cravings—carbs	−0.670	−0.760, −0.670	.08
Happiness	0.058	−0.050, 0.231	.87
Sleep	−0.030	−0.055, −0.019	.77
Other predictors			
Former WW member	0.206	0.186, 0.219	.24
Attendance at group meetings	0.234	0.193, 0.309	<.01

*Note*: Logistic regression models evaluating effect of 3‐month value of variable on likelihood of achieving 10% weight loss, with baseline value included as covariate. Result reported is parameter estimate equivalent to expected change in log odds for a 1‐unit increase in the predictor.

Abbreviation: CI, confidence interval.

## 4 DISCUSSION

The current study examined the impact of a weight management programme (WW) that included over 200 foods that did not need to be weighed, measured or tracked. This approach, which reduced the number of foods to be self‐monitored, produced an average weight loss of 7.9% across 6 months. Over 75% of participants lost 3% of initial body weight, over 60% achieved ≥5% weight loss and over 30% achieved ≥10% weight loss. Greater attendance at weekly WW workshops, decreases in hunger and reductions in fast food cravings in the first 3 months of the programme were associated with reaching a 10% weight loss at 6 months.

Weight losses in this trial are consistent with other intensive, in‐person behavioral interventions that have shown clinically significant improvements in health risk factors.^27^, ^28^ A large‐scale, multisite weight loss trial, the Diabetes Prevention Program (DPP),^29^ consisted of in‐person group sessions with intervention delivery by specialized weight loss interventionists and included detailed monitoring of dietary intake. Participants in the DPP (*n* = 1079) lost an average of 7% of their body weight, about 4–5 kg, which was associated with a 58% reduction in risk of developing type 2 diabetes. Four other 6‐month evaluations of previous WW programmes demonstrate weight losses in a similar range.^9^, ^30^, ^31^, ^32^ While aspects of the programmes studied varied (e.g., face‐to‐face and digital), they provided more traditional recommendations to self‐monitor all foods and beverages. Mean weight losses in the prior trials ranged from 3.9 to 6.6 kg at 6 months.

In addition to weight loss, participants in the current study also experienced improvements in other measured health indicators that commonly occur with weight loss, including decreased waist circumference and blood pressure, and improved aerobic stamina and flexibility. Other self‐reported health metrics that are less commonly studied, particularly in commercial programmes, also improved, including weight‐related quality of life, sleep and happiness. Self‐reported quality of life and sleep improvements seen here are consistent with those seen in other behavioral weight loss interventions, particularly among those who achieve at least 5% weight losses.^33^, ^34^, ^35^, ^36^ While less commonly measured, happiness also improved among participants over 6 months. Research has shown that happiness may be related to concurrent engagement in healthy weight‐related behaviours and improvements in happiness may also promote weight management efforts over time.^37^ These additional psychosocial and quality of life improvements have important implications for long‐term health and well‐being.

Meaningful weight losses were achieved over 6 months in the current study with an approach that significantly reduced requirements for self‐monitoring. Approaches that require detailed monitoring of all foods, including food types and amounts, may not be highly feasible or acceptable to participants due to the burden that they impose.^38^ The approach tested in this study included over 200 foods that did not require weighing, measuring or self‐monitoring and may represent a sustainable monitoring strategy. It is possible that having a large number of foods to eat without self‐monitoring helped participants manage common barriers to adherence to an energy‐reduced diet over time and thus promoted weight loss. One smaller study that examined monitoring of both food and physical activity (*n* = 42) concluded that transitioning to a simplified version of monitoring following 8 weeks of standard calorie monitoring did not negatively impact short‐term weight loss.^39^ These 6‐month data suggest that full monitoring may not be required to achieve meaningful weight reductions and improvements in other health indicators. However, studies that directly compare standard (full) monitoring to simplified monitoring approaches are needed to confirm and extend this initial finding.

Though it may be possible to use simplified versions of dietary monitoring for weight loss, this study shows that attendance at face‐to‐face sessions remains a critical part of weight loss success. Greater attendance at workshops was associated with the best weight loss outcomes, suggesting that greater exposure to intervention components and/or group support increased success. This finding is consistent with results from other behavioral interventions that have consistently shown that attendance at in‐person group meetings is associated with clinically significant weight losses.^40^, ^41^, ^42^, ^43^

In addition to session attendance, changes in several weight‐related factors were also associated with weight loss success. Decreased feelings of hunger and reduced cravings for high‐calorie, high‐fat foods over the first 3 months were associated with a greater likelihood of reaching 10% weight loss at 6 months. Allowing ad lib consumption of foods high in protein (e.g., skinless chicken breast and fish) and those high in volume and fibre (e.g., fruits and vegetables) may help manage hunger and reduce cravings.^6^, ^44^, ^45^ Several intervention studies have found that reducing energy intake by increasing intake of foods with low energy density (i.e., foods that are higher in volume and lower in calories such as fruits and vegetables) results in weight losses equal to or better than approaches that focus only on lowering fat and calories.^44^, ^45^.^46^ Future research is needed to explore these potential mechanisms.

There are several strengths of this study including excellent retention rates, high utilization of the intervention, delivery of the intervention by community‐based WW staff, and outcome assessment by separate research staff to reduce demand characteristics. There are also several limitations, most notably the lack of a comparison group. The lack of a concurrent comparison group does not permit a direct comparison of the current results to weight losses of individuals receiving a programme that used traditional (full) self‐monitoring or to a no‐treatment control group. In a meta‐regression of the weight losses of no‐treatment control groups used in 72 weight management trials, the random effect combined weight change for the control group was −0.1 kg (95% CI: −0.4, 0.1) and not statistically significantly different from zero.^47^ Therefore, it is unlikely that the effect size of the treatment would be diminished by the inclusion of a no‐treatment control group in this trial. Second, while the sample represents the general demographic of many commercial weight management programmes and clinical trials, the sample was generally highly educated, female, Caucasian and thus may have limited generalizability to other demographics. While the programme was developed and delivered by WW team members, all study participants were motivated to enrol in a research study and may have been more inclined to attend weekly meetings and adhere to the programme.

In summary, participants following a behavioral weight loss programme that promoted the unmonitored consumption of a large number of low‐energy‐dense foods lost 7.9% of initial body weight over 6 months and experienced significant improvements in blood pressure, aerobic stamina, quality of life, happiness, sleep, perceptions of hunger and reductions in cravings for high‐calorie and high‐fat foods. The promotion of numerous healthful food options that do not need to be weighed, measured or tracked may serve to reduce monitoring burden, as well as feelings of hunger and cravings, which may promote dietary adherence and weight loss over time. Future research should consider exploring adherence and other mediators, as well as to compare similar approaches to reduce monitoring burden to other forms of self‐monitoring to determine comparative effectiveness for weight loss.

## FUNDING

This study was supported by a research contract from WW to the University of North Carolina at Chapel Hill.

## CONFLICT OF INTEREST STATEMENT

DFT is a member of the Scientific Advisory Board for WW and received a research contract to conduct this study. ACW and GDF are employees and shareholders of WW. AMP was an employee of WW at the time of the study.

## References

[1] Hartmann‐Boyce J , Johns DJ , Jebb SA , Summerbell C , Aveyard P . behavioral weight management programmes for adults assessed by trials conducted in everyday contexts: systematic review and meta‐analysis. Obes Rev. 2014;15:920‐932. 25112559 10.1111/obr.12220 PMC4233997

[2] Dwyer JT , Melanson KJ , Sriprachy‐anunt U , Cross P , Wilson M . Dietary treatment of obesity In: Feingold KR, Anawalt B, Boyce A, et al., eds. Endotext. South Dartmouth (MA): MDText.com, Inc.; 2000.

[3] Butryn ML , Phelan S , Hill JO , Wing RR . Consistent self‐monitoring of weight: a key component of successful weight loss maintenance. Obesity (Silver Spring). 2007;15:3091‐3096. 18198319 10.1038/oby.2007.368

[4] Wilde MH , Garvin S . A concept analysis of self‐monitoring. J Adv Nurs. 2007;57:339‐350. 17233653 10.1111/j.1365-2648.2006.04089.x

[5] Mata J , Todd PM , Lippke S . When weight management lasts. Lower perceived rule complexity increases adherence. Appetite. 2010;54:37‐43. 19751781 10.1016/j.appet.2009.09.004

[6] Leidy HJ . Increased dietary protein as a dietary strategy to prevent and/or treat obesity. Mo Med. 2014;111(1):54‐58. 24645300 PMC6179508

[7] Rolls BJ . Dietary energy density: applying behavioral science to weight management. Nutr Bull. 2017;42:246‐253. 29151813 10.1111/nbu.12280 PMC5687574

[8] Tsai AG , Wadden TA . Systematic review: an evaluation of major commercial weight loss programs in the United States. Ann Intern Med. 2005;142:56‐66. 15630109 10.7326/0003-4819-142-1-200501040-00012

[9] Johnston CA , Rost S , Miller‐Kovach K , Moreno JP , Foreyt JP . A randomized controlled trial of a community‐based behavioral counseling program. Am J Med. 2013;126:1143.e1119‐1143.e1124. 10.1016/j.amjmed.2013.04.025 24135513

[10] Ahern AL , Wheeler GM , Aveyard P , et al. Extended and standard duration weight‐loss programme referrals for adults in primary care (WRAP): a randomised controlled trial. Lancet. 2017;389:2214‐2225. 28478041 10.1016/S0140-6736(17)30647-5 PMC5459752

[11] U.S. Department of Health and Human Services and U.S. Department of Agriculture. 2015 – 2020 Dietary Guidelines for Americans. December 2015; 8th Edition:https://health.gov/dietaryguidelines/2015/guidelines/.

[12] World Health Organization . Healthy Diet. Updated September 2015. http://www.who.int/mediacentre/factsheets/fs394/en/. Accessed March 9, 2018

[13] Mifflin MD , St Jeor ST , Hill LA , Scott BJ , Daugherty SA , Koh YO . A new predictive equation for resting energy expenditure in healthy individuals. Am J Clin Nutr. 1990;51:241‐247. 2305711 10.1093/ajcn/51.2.241

[14] Centers for Disease Control and Prevention . National Health and Nutrition Examination Survey (NHANES): Anthropometry Procedures Manual. January 2007.

[15] ATS statement: guidelines for the six‐minute walk test. Am J Respir Crit Care Med. 2002;166:111‐117. 12091180 10.1164/ajrccm.166.1.at1102

[16] King MB , Judge JO , Whipple R , Wolfson L . Reliability and responsiveness of two physical performance measures examined in the context of a functional training intervention. Phys Ther. 2000;80:8‐16. 10623956

[17] American College of Sports Medicine . ACSM's Guidelines for Exercise Testing and Prescription. 9th ed. Philadelphia: Wolters Kluwer/Lippincott Williams & Wilkins Health; 2014.

[18] Flint A , Raben A , Blundell JE , Astrup A . Reproducibility, power and validity of visual analogue scales in assessment of appetite sensations in single test meal studies. Int J Obes Relat Metab Disord. 2000;24:38‐48. 10702749 10.1038/sj.ijo.0801083

[19] Womble LG , Wadden TA , Chandler JM , Martin AR . Agreement between weekly vs. daily assessment of appetite. Appetite. 2003;40:131‐135. 12781162 10.1016/s0195-6663(02)00170-8

[20] Tronieri JS , Wadden TA , Walsh O , et al. Effects of liraglutide on appetite, food preoccupation, and food liking: results of a randomized controlled trial. Int J Obes (Lond). 2020;44:353‐361. 30926955 10.1038/s41366-019-0348-6 PMC6766432

[21] White MA , Whisenhunt BL , Williamson DA , Greenway FL , Netemeyer RG . Development and validation of the food‐craving inventory. Obes Res. 2002;10:107‐114. 11836456 10.1038/oby.2002.17

[22] Buysse DJ , Reynolds CF 3rd , Monk TH , Berman SR , Kupfer DJ . The Pittsburgh Sleep Quality Index: a new instrument for psychiatric practice and research. Psychiatry Res. 1989;28:193‐213. 2748771 10.1016/0165-1781(89)90047-4

[23] Carpenter JS , Andrykowski MA . Psychometric evaluation of the Pittsburgh Sleep Quality Index. J Psychosom Res. 1998;45:5‐13. 9720850 10.1016/s0022-3999(97)00298-5

[24] Hills P , Argyle M . The Oxford Happiness Questionnaire: a compact scale for the measurement of psychological well‐being. Personality and Individual Differences. 2002;33:1073‐1082.

[25] Kolotkin RL , Crosby RD , Kosloski KD , Williams GR . Development of a brief measure to assess quality of life in obesity. Obes Res. 2001;9:102‐111. 11316344 10.1038/oby.2001.13

[26] Thomas JG , Raynor HA , Bond DS , et al. Weight loss in Weight Watchers Online with and without an activity tracking device compared to control: a randomized trial. Obesity (Silver Spring). 2017;25:1014‐1021. 28437597 10.1002/oby.21846

[27] Delahanty LM , Nathan DM . Implications of the diabetes prevention program and Look AHEAD clinical trials for lifestyle interventions. J Am Diet Assoc. 2008;108:S66‐S72. 18358260 10.1016/j.jada.2008.01.026 PMC2726971

[28] Elmer PJ , Obarzanek E , Vollmer WM , et al. Effects of comprehensive lifestyle modification on diet, weight, physical fitness, and blood pressure control: 18‐month results of a randomized trial. Ann Intern Med. 2006;144:485‐495. 16585662 10.7326/0003-4819-144-7-200604040-00007

[29] Diabetes Prevention Program Research Group . The Diabetes Prevention Program (DPP): description of lifestyle intervention. Diabetes Care. 2002;25:2165‐2171. 12453955 10.2337/diacare.25.12.2165 PMC1282458

[30] Heshka S , Greenway F , Anderson JW , et al. Self‐help weight loss versus a structured commercial program after 26 weeks: a randomized controlled study. Am J Med. 2000;109:282‐287. 10996578 10.1016/s0002-9343(00)00494-0

[31] Thomas JG , Raynor HA , Bond DS , et al. Weight loss and frequency of body‐weight self‐monitoring in an online commercial weight management program with and without a cellular‐connected 'smart' scale: a randomized pilot study. Obes Sci Pract. 2017;3:365‐372. 29259794 10.1002/osp4.132 PMC5729493

[32] Truby H , Baic S , DeLooy A , et al. Randomised controlled trial of four commercial weight loss programmes in the UK: initial findings from the BBC "diet trials". Bmj. 2006;332:1309‐1314. 16720619 10.1136/bmj.38833.411204.80 PMC1473108

[33] Kolotkin RL , Crosby RD , Williams GR , Hartley GG , Nicol S . The relationship between health‐related quality of life and weight loss. Obes Res. 2001;9:564‐571. 11557837 10.1038/oby.2001.73

[34] Sarwer DB , Moore RH , Diewald LK , et al. The impact of a primary care‐based weight loss intervention on the quality of life. Int J Obes (Lond). 2013;37:S25‐S30. 23921778 10.1038/ijo.2013.93 PMC3786773

[35] Holland‐Carter L , Tuerk PW , Wadden TA , et al. Impact on psychosocial outcomes of a nationally available weight management program tailored for individuals with type 2 diabetes: results of a randomized controlled trial. J Diabetes Complications. 2017;31:891‐897. 28319001 10.1016/j.jdiacomp.2017.01.022

[36] Kolotkin RL , Norquist JM , Crosby RD , et al. One‐year health‐related quality of life outcomes in weight loss trial participants: comparison of three measures. Health Qual Life Outcomes. 2009;7:53. 19505338 10.1186/1477-7525-7-53 PMC2700089

[37] Cook E , Chater A . Are happier people, healthier people? The relationship between perceived happiness, personal control, BMI and health preventive behaviours. International Journal of Health Promotion and Education. 2010;48:58‐64.

[38] Solbrig L , Jones R , Kavanagh D , May J , Parkin T , Andrade J . People trying to lose weight dislike calorie counting apps and want motivational support to help them achieve their goals. Internet Interv. 2017;7:23‐31. 28286739 10.1016/j.invent.2016.12.003 PMC5332530

[39] Helsel DL , Jakicic JM , Otto AD . Comparison of techniques for self‐monitoring eating and exercise behaviors on weight loss in a correspondence‐based intervention. J Am Diet Assoc. 2007;107:1807‐1810. 17904942 10.1016/j.jada.2007.07.014

[40] Wadden TA , West DS , Neiberg RH , et al. One‐year weight losses in the Look AHEAD study: factors associated with success. Obesity (Silver Spring). 2009;17:713‐722. 19180071 10.1038/oby.2008.637 PMC2690396

[41] Wing RR , Hamman RF , Bray GA , et al. Achieving weight and activity goals among diabetes prevention program lifestyle participants. Obes Res. 2004;12:1426‐1434. 15483207 10.1038/oby.2004.179 PMC2505058

[42] Fabricatore AN , Wadden TA , Moore RH , Butryn ML , Heymsfield SB , Nguyen AM . Predictors of attrition and weight loss success: results from a randomized controlled trial. Behav Res Ther. 2009;47:685‐691. 19497559 10.1016/j.brat.2009.05.004 PMC2713356

[43] Johnston CA , Moreno JP , Hernandez DC , et al. Levels of adherence needed to achieve significant weight loss. Int J Obes (Lond). 2019;43:125‐131. 30301963 10.1038/s41366-018-0226-7

[44] Ello‐Martin JA , Roe LS , Ledikwe JH , Beach AM , Rolls BJ . Dietary energy density in the treatment of obesity: a year‐long trial comparing 2 weight‐loss diets. Am J Clin Nutr. 2007;85:1465‐1477. 17556681 10.1093/ajcn/85.6.1465 PMC2018610

[45] Buckland NJ , Camidge D , Croden F , et al. A low energy‐dense diet in the context of a weight‐management program affects appetite control in overweight and obese women. J Nutr. 2018;148:798‐806. 30053284 10.1093/jn/nxy041 PMC6054218

[46] Vadiveloo M , Parker H , Raynor H . Increasing low‐energy‐dense foods and decreasing high‐energy‐dense foods differently influence weight loss trial outcomes. Int J Obes (Lond). 2018;42:479‐486. 29406521 10.1038/ijo.2017.303 PMC5902316

[47] Waters L , George AS , Chey T , Bauman A . Weight change in control group participants in behavioral weight loss interventions: a systematic review and meta‐regression study. BMC Med Res Methodol. 2012;12:120. 22873682 10.1186/1471-2288-12-120 PMC3499351

